# Gender Differentiation in Indirect Self-Destructiveness and Suicide Attempt Methods (Gender, Indirect Self-Destructiveness, and Suicide Attempts)

**DOI:** 10.1007/s11126-013-9283-1

**Published:** 2013-12-04

**Authors:** Konstantinos Tsirigotis, Wojciech Gruszczyński, Marta Tsirigotis-Maniecka

**Affiliations:** 1 Department of Psychology, Jan Kochanowski University, Piotrków Trybunalski Branch, Słowackiego 114/118 Str, 97-300 Piotrków Trybunalski, Poland; 2 Psychosocial Rehabilitation Department, The Medical University of Lodz, Lodz, Poland; 3 Organic and Pharmaceutical Technology Group, Chemistry Department, Wroclaw University of Technology, Wroclaw, Poland

**Keywords:** Indirect self-destructiveness, Gender, Sex, Suicide attempt methods

## Abstract

The objective of this study is to examine the gender (sex) differentiation of indirect self-destructiveness and its manifestations as well as its relationships with suicide attempt methods in females and males. The study was conducted among 147 persons (114 females, 33 males) who attempted suicide. The research instrument was the polish version of the Chronic Self-Destructiveness Scale including Transgression and Risk, Poor Health Maintenance, Personal and Social Neglects, Lack of Planfulness, and Helplessness and Passiveness in the face of problems. Differences testing and correlation analyses were applied. Females scored higher on poor health maintenance and males scored significantly higher on personal and social neglects, lack of planfulness, and helplessness. Noteworthy is that the intensity of indirect self-destructiveness in females reached the same magnitude as in males. A number of statistically significant correlations were found between indirect self-destructiveness, or its manifestations, and the methods of suicide attempt in the two groups. Among these categories, the highest contribution was of helplessness and passiveness (both of groups), poor health maintenance (males), and personal and social neglects (females). Results of this study can be useful in the therapeutic efforts and prevention of not only indirectly self-destructive behaviours but also possible suicide attempts. Both preventive and therapeutic activities can take into account the specificity of those phenomena resulting from one’s sex/gender. It is important to adapt preventive and therapeutic measures to psychological (personal) features that arise from an individual’s sex/gender.

## Introduction

Suicide is an issue important individually or intrapsychically as well as socially. In the individual/intrapsychic dimension it is an expression and, simultaneously, a consequence of human suffering while in the social dimension it causes suffering to those close to a suicide and loss to the society deprived of a valuable human being and benefit to be derived from him or her.

In general, suicides are among main causes of death worldwide with mortality due to suicidal acts accounting for about 2 % and suicide attempts being a serious risk factor of a committed suicide [1]. The number of suicides increases worldwide, especially in the population of young people and adult males [2, 3]. Moreover, numbers of suicides committed using violent methods, such as hanging, also grow, which suggests that those more lethal methods contribute to an increased degree of suicide risk [4]. An attempted suicide not resulting in death is among the strongest clinical predictors of committed suicide, which is reflected in recurrence of suicide attempts [5, 6].

Suicide is most commonly perceived in terms of direct self-destructiveness. However, suicide, and (non-fatal) suicide attempts in particular, can also be regarded as a manifestation or effect of indirect self-destructiveness.

Chronic or indirect self-destructiveness is described as a generalised tendency to undertake behaviours increasing the likelihood of negative and decreasing the likelihood of positive consequences for a subject [7]. Indirect self-destructiveness is also defined as behaviours whose likely negative effect is intermediated by additional factors while the relationship between behaviour and harm is perceived as likely. Indirect self-destructiveness understood in such a way comprises both taking and abandoning specific actions; it concerns getting into hazardous and increased-risk situations (active form) or neglecting one’s safety or health (passive form). Whereas an acute self-destructive behaviour entails a conscious and purposeful intention to undertake painful and harmful acts against oneself, sometimes with the intention of killing oneself, chronic/indirect self-destructiveness is related to acts performed during a certain period of time and in some situations with the subject being unaware of or disregarding their long-term harmful effects [8, 9].

It is not a coincidence that indirect-self destructiveness is referred to as “slow” or „lingering” suicide.

Results of research indicate that females prefer the so-called “soft” and less effective suicide methods in contrast to males who prefer the so-called “hard” and more effective ones. That, among others, explains the so-called “gender paradox” in suicides: although relatively more females attempt suicide, relatively more males die by suicide [10–13]. Thus, females are considered “attempters” and “survivors” of suicide attempts [13].

Research reports reveal that males display more self-destructive behaviours. Most studies, however, concerned direct self-destructiveness (self-mutilation, self-inflicted injuries, suicide attempts, suicides) or single, isolated manifestations of indirect self-destructiveness. In world literature there are almost no studies into the gender (sex) differentiation of indirect self-destructiveness intensity as a generalised tendency considered in a comprehensive, holistic manner. One of the few research studies into the gender (sex) differentiation of indirect self-destructiveness stated that indirect self-destructiveness, as a generalised behavioural tendency, is more intense in males than in females [14].

It is a well-known fact that females and males differ from each other not only physically or in their external appearance but, first and foremost, in the way they construe and experience the world and function psychologically.

Question: Do they also differ in the intensity of indirect self-destructiveness (and its manifestations) in connection with suicide attempt methods?

The objective of this study is to examine the gender (sex) differentiation of indirect self-destructiveness and its manifestations as well as its relationships with suicide attempt methods in females and males.

## Methods

The study is part of a more extensive research project whose results were already published in part and thus the applied methods are similar [15].

## Participants

The study was conducted among persons who attempted suicide using different methods and were admitted to hospital thereafter. The examinations were carried out after the patients had completed their medical treatment. The study population comprised 147 participants (114 females and 33 males) between 23 and 33 years of age.

When the treatment of the somatic effects of suicide attempt had been completed, the patients underwent standard psychiatric and psychological examinations (mostly at mental health centres) conducted by psychiatrists and clinical psychologists. The findings revealed no psychotic disorders or mental retardation among the study population. The examination was performed using first a structured, self-reported questionnaire developed by the author as well as the medical records of the subjects. Then the CS-DS questionnaire was administered to the subjects by well-trained and experienced clinical psychologists. The researchers were present during the examination to help the respondents with the questionnaire items if necessary. The entire research project lasted approximately 12 months.

The examination was anonymous and the participation was voluntary. It is worth noting that there were no refusals. Informed consent, according to the Helsinki declaration recommendations, was obtained from each participant and the health centre management granted permission to perform the study on their patients.

## Materials

In order to examine indirect self-destructiveness and its manifestations, the Polish version of “chronic self-destructiveness scale” by Kelley (CS-DS), as adapted by Suchańska, was administered. Kelley’s scale assessing chronic (indirect) self-destructiveness as a generalised tendency includes four categories of behaviour: carelessness, poor health maintenance, evidence of transgression, and lack of planfulness. The ultimate version consists of an internal, coherent set of 52 items [7].

Both the original scale and its Polish adaptation are characterised by high reliability and validity. For the original scale, the reliability (internal consistency, Cronbach’s alpha) ranged from 0.90 to 0.98 [7]. In the Polish adaptation, the values varied from 0.799 to 0.811 for reliability (Cronbach’s alpha) and from 0.752 to 0.861 for content validity [9].

The Polish scale comprises the following categories: Transgression and Risk (A1; e.g. I use or have used street drugs; An occasional fight makes a guy more of a man), Poor Health Maintenance (A2; e.g. I have a complete physical examination once a year; I always do what my doctor or dentist recommends), Personal and Social Neglects (A3; e.g. I usually meet deadlines with no trouble; I am frequently late for important things), Lack of Planfulness (A4; e.g. I just don’t know where my money goes; I hate any kind of schedule or routine), and Helplessness and Passiveness in the face of problems (A5; e.g. Sometimes I don’t seem to care what happens to me; It’s easy to get a raw deal from life), the scores for which sum up to one global score for indirect self-destructiveness [8, 9].

Descriptive and statistical inference methods were used in the statistical analysis of the quantitative data. To characterise the average value for quantitative traits, the arithmetic mean (M) was calculated, whereas standard deviation (SD) was assumed as a measure of dispression. The consistency of quantitative features distribution was evaluated using the Shapiro-Wilk test. Because of the lack of consistency of the distributions of the dependent variables with the normal distribution, in the statistical analysis of the obtained results non-parametric statistics were used.

In order to examine the differences between men and women in CS-DS the Mann-Whitney *U* test was applied. The correlation analyses were used to examine the relationship between the different manifestations of indirect self-destructiveness and the particular methods of suicide attempts in the two groups (Kendall Tau, *τ*); *p* ≤ 0.05 was considered significant. All the calculations were made using *Statistica PL 10.0* [16].

## Results

**Fig. 1:**
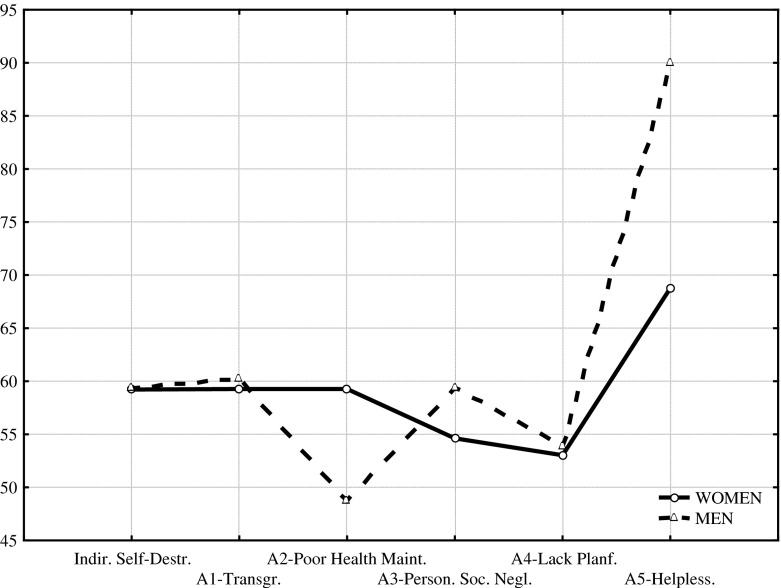
The females and males suicides profiles in the CS-DS

Table 1 and Fig. 1 present comparison of CS-DS scores achieved by males and females after suicide attempts (the Mann-Whitney *U* test).

**Table 1:** Comparison of results (M ± SD) obtained by females and males suicides in CS-DS

Variables	Women		Men		Significance	
	M	SD	M	SD	U	*p*
Indirect Self-Destructiveness	153.974	24.958	154.273	12.809	1791.000	ns
A1-Transgression, Risk	53.342	11.981	54.273	8.694	1651.500	ns
A2-Poor Health Maintenance	32.605	6.957	26.818	6.507	1143.000	0.00004
A3-Personal and Social Neglects	32.789	7.129	35.636	4.506	1318.500	0.008
A4-Lack of Planfulness	21.211	5.517	24.273	4.564	1359.000	0.004
A5-Helplessness, Passiveness	9.000	2.101	15.316	0.968	985.500	*p* < 0.00 001

As it can be seen, statistically significant differences were found for four indicators: A2—Poor Health Maintenance, A3—Personal and Social Neglects, A4—Lack of Planfulness, and A5—Helplessness, Passiveness in the face of problems/difficulties. Females scored significantly higher on Poor Health Maintenance (A2) and males—on the other categories of indirectly self-destructive behaviours: Personal and Social Neglects (A3), Lack of Planfulness (A4), and Helplessness (A5).

Tables 2 and 3 show correlation coefficients (the Kendall tau, *τ*) among CS-DS indicators and specific suicide attempt methods in the group of males and in the group of females respectively.

**Table 2:** Correlations between manifestations of indirect self-destructiveness and suicide attempt methods in the males group

Variable	Drug overdose	Wrist cutting	Hanging	Jumping from a height	Asphyxia	Poisoning	Walking into traffic	Recurrence
Indirect Self-Destructiveness	0.714				−0.778			0.583
	*p* = 0.00002	ns	ns	ns	*p* = 0.0002	ns	ns	*p *= 0.001
Transgression, Risk (A1)	0.385				−0.444			0.667
	*p* = 0.03	ns	ns	ns	*p* = 0.03	ns	ns	*p* = 0.0002
Poor Health Maintenance (A2)	0.571	−0.529	0.586	−0.800	−0.778			0.667
	*p* = 0.0007	*p* = 0.01	*p *= 0.0005	*p* = 0.004	*p* = 0.0002	ns	ns	*p *= 0.0002
Personal and Social Neglects (A3)		−0.889		0.702	−0.774			0.652
	ns	*p *= 0.00002	ns	*p *= 0.0003	*p* = 0.000002	ns	ns	*p* = 0.0005
Lack of Planfulness (A4)		−0.889			−0.444			
	ns	*p* = 0.00001	ns	ns	*p* = 0.03	ns	ns	ns
Helplessness and Passiveness (A5)	0.600	0.749	−0.826	0.586				
	*p *= 0.004	*p* = 0.00003	*p* = 0.00001	*p* = 0.005	ns	ns	ns	ns

**Table 3:** Correlations between manifestations of indirect self-destructiveness and suicide attempt methods in the females group

Variable	Drug overdose	Wrist cutting	Hanging	Jumping from a height	Asphyxia	Poisoning	Walking into traffic	Recurrence
Indirect Self-Destructiveness	0.446	0.192				0.622		0.181
	*p* = 0.00001	*p *= 0.02	ns	ns	ns	*p* = 0.02	ns	*p* = 0.04
Transgression, Risk (A1)	0.444	0.263				−0.611		0.306
	*p* = 0.00002	*p* = 0.003	ns	ns	ns	*p* = 0.02	ns	*p* = 0.0008
Poor Health Maintenance (A2)	0.547							
	*p* = 0.0000002	ns	ns	ns	ns	ns	ns	ns
Personal and Social Neglects (A3)				−0.286	0.892	0.778	0.892	−0.241
	ns	ns	ns	*p* = 0.02	*p* = 0.0009	*p* = 0.005	*p* = 0.0009	*p *= 0.009
Lack of Planfulness (A4)	0.217					0.889		
	*p* = 0.03	ns	ns	ns	ns	*p* = 0.001	ns	ns
Helplessness and Passiveness (A5)				0.299	0.806	0.857	0.806	
	ns	ns	ns	*p* = 0.02	*p *= 0.008	*p* = 0.0001	*p* = 0.008	ns

As it can be seen, there are many statistically significant correlation coefficients but there are certain differences between correlations found in the group of males and in the group of females: some of them occur in only one group while others, although present in both, have a different sign (plus or minus) in each of them.

In the group of males the highest correlation coefficient was observed between Helplessness (A5) and hanging with a negative sign (−0.826); while in the group of females Helplessness (A5) was also characterized by the highest correlation coefficient—but with poisoning (0.857). In the group of males, Helplessness (A5) correlated mainly with suicide methods that are generally more common whereas in females—with less common ones.

In the group of males, out of all CS-DS indicators, the largest number of correlations with suicide attempt methods occurred for Poor Health Maintenance (A2) with those relationships being noted only in the group of males; while in the group of females the largest number of correlations with suicide attempt methods was found for Personal and Social Neglects (A3).

In turn, out of all suicide attempt methods, in the group of males the largest number of correlations with CS-DS indicators occurred for asphyxia and those were negative correlations; whereas in the group of females the largest number of correlations with CS-DS indicators was detected for poisoning and those were mostly positive correlations with the only negative one found for Transgression (A1).

## Discussion

Since literature offers a scarce number of studies into relationships between indirect self-destructiveness and its manifestations and suicide attempt methods, especially into their gender (sex) differentiation, it will be difficult to refer to results of other studies.

Based on earlier conducted research it was found that the intensity of indirect self-destructiveness as a generalised tendency and the intensity of its specific manifestations (Transgression and Risk, Poor Health Maintenance, Personal and Social Neglects, Lack of Planfulness, and Helplessness, Passiveness in the face of problems/difficulties) were significantly higher in individuals who attempted than in those who did not attempt suicide [17]. That can indicate that indirect self-destructiveness may be a predictor or risk factor of committing (attempting) suicide.

It is worth pondering over the fact that although in the general population (with no suicide attempts) the intensity of indirect self-destructiveness, as a generalised behavioural tendency, is higher in males [14], the received results reveal that it is equal in males and females who attempted suicide. Could females who attempted suicide “catch up” with males in respect of indirect self-destructiveness? It is important insofar as in the aspect of attempted suicides (and maybe committed suicides), especially in the case of females, increased indirect self-destructiveness may be a risk factor and alarm sign of suicide attempts.

In the studied group of individuals who attempted suicide there were statistically significant differences in results between females and males in four categories of indirectly self-destructive behaviours. Females scored higher on poor health maintenance (A2) and males scored significantly higher on three categories: personal and social neglects (A3), lack of planfulness (A4), and helplessness (A5). Results were similar in the general population (of individuals who did not attempt suicide) with the difference being that in the general population males scored significantly higher also on poor health maintenance (A2) [14]. As it can be seen, the result is opposite in the group of females who attempted suicide. It is symptomatic that males after suicide attempts neglect their health to a degree lower than females after suicide attempts and males with no suicide attempt history but similarly to females who did not attempt suicide—thus to a very low degree; whereas females after suicide attempts poorly maintain their health to the highest degree as compared to the other groups (i.e. males who attempted suicide as well as females and males who did not attempt suicide). Therefore, it can be assumed that poor health maintenance in females may be an alarming sign as regards suicide attempts. That would be yet another risk indicator of suicide (attempt) in females. On the other hand, could a low degree of poor health maintenance in males, paradoxically, be a prodrome sign of suicide attempt?

As already mentioned, a great number of statistically significant correlations were found between indirect self-destructiveness manifestations and suicide attempt methods in both the group of females and the group of males. To avoid repeating a description of previous research results [15], the study will mainly focus on those relationships that, in our opinion, contribute further to the understanding of relationships between indirect self-destructiveness and its manifestations and gender (sex) and suicide attempts.

In the group of males, the largest number of correlations with specific suicide attempt methods occurred for poor health maintenance (A2) with those relationships being noted only in the group of males. Moreover, it is worth emphasizing that it was the sole category of indirectly self-destructive behaviours where males achieved lower scores than females after suicide attempts and similar scores to females with no suicide attempt history (i.e. low); hence, it can be assumed that that category of indirectly self-destructive behaviours plays an important role in attempting suicide by males. Thus, the already posed question arises again: could a low degree of health neglecting in males, paradoxically, be a prodrome sign of suicide attempt? The question is important insofar as that category of indirect self-destructiveness correlates with recurrence of suicide attempts, which is a high risk factor of committed suicide [15, 18–20].

In turn, out of suicide attempt methods, the largest number of correlations with specific indirect self-destructiveness categories occurred, in the group of males, for asphyxia. It does not come as a surprise considering the fact that males use that method more commonly than females; asphyxia is characterized by a moderate degree of body integrity breach and a moderate degree of self-aggression (higher, however, than for poisoning), and a high degree of effectiveness [13]. The negative sign of correlation coefficient may suggest that such a suicide attempt method in males may not necessarily be associated with indirect self-destructiveness (may not result from indirectly self-destructive tendencies) but may be perceived by them as a way out or solution; such a conjecture may be supported by observing a problem-focus coping attitude to problems in males [cf. 21–23].

Among suicide attempt methods in males, the second largest number of coefficients of correlation with manifestations of indirect self-destructiveness is observed for wrist cutting. Correlations between wrist cutting and manifestations (categories) of indirect self-destructiveness are mostly negative (only helplessness, A5, correlates positively); it is a method characterized by low effectiveness and is less commonly chosen by males.

Hanging in males is related to poor health maintenance (A2), which should not come as a surprise as a suicide attempt is a manifestation of disregarding (giving up) life as such and not only one’s health; it should be emphasized that that method is among the most effective and is more commonly used by males. Hanging and asphyxia are methods applied most frequently by individuals who died as a result of a consecutive suicide attempt; they are high risk factors for a recurrent fatal suicide attempt [24].

Jumping from a height negatively correlates with poor health maintenance (A2) in males probably due to the fact that it is a highly effective and self-aggressive method; it is quite possible that in that case good or poor health maintenance is less important than the final outcome, i.e. death (males’ suicide attempts are more frequently fatal).

Asphyxia in males negatively correlates with almost all indicators (categories) of indirect self-destructiveness; it is that very method that is mainly applied by males.

Recurrence of suicide attempt in the group of males is related to poor health maintenance (A2) and personal and social neglects (A3). It is difficult to talk about health maintenance in the case of suicide attempt, especially because such attempts are more often fatal in males. We will return to relationships between that indicator and personal and social neglects further in the study.

In turn, in the group of females the largest number of correlations with specific suicide attempt methods occurred for personal and social neglects (A3), which were less intense in females than in males; hence, it can be assumed that that category of indirectly self-destructive behaviours plays and important role in attempting suicide by females. Attention should be given to the negative correlation coefficient between that category and recurrence of suicide attempts; maybe caring (for oneself and others) protects females from recurrence of suicide attempts. Furthermore, it is worth noting that personal and social neglects have coefficients of correlation with some suicide attempt methods with different signs in the group of males and in the group of females (plus, minus), to which we will return further in the study. In turn, out of all suicide attempt methods, the largest number of correlations with specific indirect self-destructiveness categories was observed for poisoning; females apply that method more often than males. Poisoning is characterised by the lowest degree of body integrity breach and self-aggression among all suicide attempt methods as well as a moderate degree of effectiveness [13].

Relationships between lack of planfulness (A4) and pharmacological drugs in females may suggest that an attempt was made using what was within one’s reach. It should be kept in mind that abuse of pharmacological drugs is among the least effective suicide methods and is characterised by the lowest self-aggression degree. Therefore, it can be assumed that, in females, lack of planfulness predisposes to non-fatal suicide attempts that are rather a “manifestation” of problems or a cry for help.

Wrist cutting in females is associated with indirect self-destructiveness as a generalised behavioural tendency. It is a method more commonly used by females, also characterised by a low degree of effectiveness and self-aggression. Maybe that is why it negatively correlates with poor health maintenance (A2), personal and social neglects (A3), and lack of planfulness (A4) in males who apply that method less commonly than females.

Poisoning is a suicide attempt method used solely by females and it is only in that group that it correlates with indirect self-destructiveness manifestations. That method is characterised by the lowest body integrity breach degree and thus, the lowest degree of self-aggression [13, 15, 25, 26]. The relationship between poisoning and lack of planfulness (A4) may result from the fact that females tried to poison themselves with what they had within their reach. In turn, the degree of body integrity breach seems to be quite important in choosing a suicide attempt method. Research indicates that females choose methods that distort their external appearance to a lower degree [12, 27]. That indicates gender determinants resulting not only from psychopathology or “suicidology” but also from (gender) psychology: even when facing death (or maybe even an attempt at it), females are interested in aesthetic qualities and their own appearance [13].

Walking into traffic shows relationships (correlates), only in the group of females, with personal and social neglects (A3) and helplessness (A5). The issue of helplessness in suicide attempts is quite clear: suicide is an expression and maybe also an effect of a human’s helplessness in a situation he or she face [cf. 15, 19, 28]. A little more attention should be given to the relationship between that method and personal and social neglects (A3). It may be the case that in that way an attempter directly involves other people (the driver and possibly passengers and passers-by) in his or her problems and forcibly “burdens” those people with them. Quite possibly it is a desperate attempt at establishing relations with other people and maybe a desperate “cry for help”, especially as that suicide attempt method also correlates with helplessness (as mentioned above).

Helplessness (A5) significantly contributes to suicide attempts made by both males and females [cf. 15]. However, the way in which that contribution is “distributed” is interesting. In males it is associated with more common methods (although males less frequently attempt and more frequently commit suicide) such as pharmacological drugs abuse, wrist cutting, hanging, and jumping from a height whereas in females—with less common methods (although females more often attempt suicide): jumping from a height, asphyxia, poisoning, and walking into traffic.

Attention should be paid to relationships occurring with different signs (plus, minus) in the group of males and in the group of females: personal and social neglects (A3) and jumping from a height, asphyxia, and recurrence. That category of indirectly self-destructive behaviours was more intense in males.

Personal and social neglects positively correlate with jumping from a height in males and negatively correlate with that method in females. It is possible that in that case males display extreme lack of care for all the things important to them; that may be supported by the fact that after a jump, when falling, an individual has little influence on what happens to him or her. Motivation, however, in females may be different in the case of jumping: making or abandoning such a decision in females may be influenced by an attitude of caring for everyone and everything that is typical of females; after all, it is females who take the most care e.g. of a child from his or her birth (at least at the beginning of the child’s life) and they are also brought up in the spirit of “caring” before they become mothers.

Another suicide method with which personal and social neglects correlate differently in males and females is asphyxia which correlates positively in females and negatively in males. At this point it should be reminded that that method correlates negatively with almost all indirect self-destructiveness categories in the group of males although it is a method they more commonly use. Thus, it can be assumed that their motivation to make such a suicide attempt is not necessarily indirectly self-destructive but is of another nature: e.g. an escape from a problem or suffering, and maybe they perceive such a method as a way out? In turn, the positive relationship of that method in females (less commonly used by them) may indicate that they completely give up all things in the personal and social dimension, which does not come as a surprise as suicide is tantamount to giving up life as such.

Another indicator with which personal and social neglects are differently associated in the group of males (positively) and in the group of females (negatively) is recurrence. It can be assumed that determination of a suicide (a male repeating his suicide attempt) makes him give up almost everything as he gives up life. In females, on the other hand, the negative relationship with personal and social neglects can mean that care for oneself and concern for others may protect females from recurrence of suicide attempts.

## Conclusions

The intensity of indirect self-destructiveness, as a generalised behavioural tendency, in females who attempted suicide achieved the level observed in males who attempted suicide, which can be a warning sign of suicide attempts in females.

Poor health maintenance can also be a warning sign in females who scored higher on them than the group of males.

Results of this study can be useful in the prevention of not only indirectly self-destructive behaviours but also possible suicide attempts. They can also be applied in therapy work with individuals who display such tendencies or made an attempt on their own life [compare cf. 6, 29–31]. Both preventive and therapeutic activities can take into account the specificity of those phenomena resulting from one’s sex/gender. It is important to adapt preventive and therapeutic measures to psychological (personal) features that arise from an individual’s sex/gender.

### Limitations

The authors realise that the study does not exhaust all aspects of that complicated issue. It did not examine relationships between gender and suicide attempt methods, which seems a very interesting and significant issue that may constitute the object of further research. In turn, a special area of therapeutic activities can be the sex/gender differentiation of poor or insufficiently developed coping abilities that are common in indirect self-destructiveness.
